# Cerebral Embolic Protection Devices During Transcatheter Aortic Valve Replacement: A Meta-analysis of Randomized Controlled Trials

**DOI:** 10.1016/j.jscai.2023.101031

**Published:** 2023-08-25

**Authors:** Rohin K. Reddy, Yousif Ahmad, Ahran D. Arnold, James P. Howard

**Affiliations:** a Cardiovascular Trials and Epidemiology Section, National Heart and Lung Institute, Imperial College London, London, United Kingdom; b Section of Cardiovascular Medicine, Yale University, New Haven, Connecticut

**Keywords:** transcatheter aortic valve replacement, cerebral embolic protection devices, stroke, meta-analysis, randomized controlled trial

## Abstract

**Background:**

Stroke is a feared complication of transcatheter aortic valve replacement (TAVR), which embolic protection devices (EPDs) may mitigate. This systematic review and meta-analysis synthesized randomized controlled trials (RCTs) to evaluate the effect of EPDs in TAVR.

**Methods:**

All RCTs comparing EPDs with control during TAVR were systematically identified. Prespecified primary end points were all stroke, disabling stroke, nondisabling stroke, and all-cause mortality. Safety and neuroimaging parameters were assessed. Sensitivity analyses were stratified by EPD type. Study registration was a priori (CRD42022377939).

**Results:**

Eight trials randomizing 4043 patients were included. There was no significant difference between EPDs and control for all stroke (relative risk [RR], 0.88; 95% CI, 0.65-1.18; *P* = .39; *I*^2^ = 0%), disabling stroke (RR, 0.67; 95% CI, 0.31-1.46; *P* = .32; *I*^2^ = 8.6%), nondisabling stroke (RR, 0.99; 95% CI, 0.71-1.40; *P* = .97; *I*^2^ = 0%), or all-cause mortality (RR, 0.87; 95% CI, 0.43-1.78; *P* = .71; *I*^2^ = 2.3%). There were no differences in safety end points of bleeding, vascular complications, or acute kidney injury. EPDs did not result in differences in total lesion volume or the number of new lesions. The Sentinel EPD significantly reduced the risk of disabling stroke (RR, 0.42; 95% CI, 0.20-0.88; *P* = .022; *I*^2^ = 0%) but did not affect all stroke, nondisabling stroke, or all-cause mortality.

**Conclusions:**

The totality of randomized data for EPDs during TAVR demonstrated no safety concerns or significant differences in clinical or neuroimaging end points. Analyses restricted to the Sentinel EPD demonstrated large, clinically meaningful reductions in disabling stroke. Ongoing RCTs may help validate these results.

## Highlights

• Meta-analysis of 8 randomized trials enrolling 4043 patients.
• Embolic protection did not alter efficacy, safety, or neuroimaging outcomes.
• The Sentinel device resulted in lower rates of disabling stroke.

## Central Illustration

**Figure:**
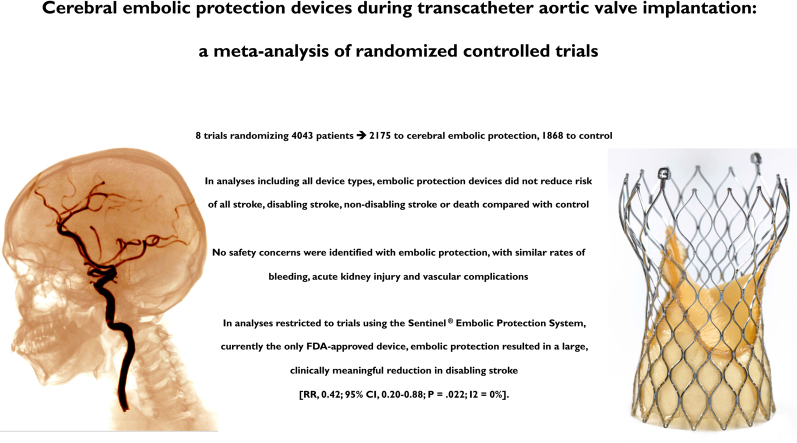

## Introduction

Transcatheter aortic valve replacement (TAVR) was initially considered for patients unsuitable for surgical aortic valve replacement.^1^ Randomized controlled trials (RCTs) have since demonstrated favorable safety and efficacy profiles across the full spectrum of surgical risk.^2^, ^3^, ^4^, ^5^, ^6^, ^7^ Therefore, reducing procedural complications remains paramount given the extension of TAVR to low-risk patient populations. Encouragingly, contemporary registry studies of TAVR show reductions in mortality and length of hospital stay over time.^8^

Stroke is a feared complication of TAVR, conferring disabling morbidity and mortality. Patients consider stroke a worse outcome than death.^9^ Despite improvements in device technology and refinements in procedural technique, stroke rates remain largely inert since the advent of TAVR at ≈2%.^8^ Embolization likely represents the dominant mechanism whereby particulate valvular or vascular wall tissue and atherosclerotic plaque may dislodge after instrumentation of the aorta and subsequent valve deployment. Cerebral embolic protection devices (EPDs) were developed on the predicate that minimizing procedural embolization by capturing or deflecting debris from carotid and vertebral arteries would reduce stroke during TAVR.

We previously synthesized RCTs investigating cerebral EPDs as a method of mechanical stroke prophylaxis illustrating no benefit of EPDs on clinical outcomes or neuroimaging parameters.^10^ Three new RCTs have since been published,^11^, ^12^, ^13^ with PROTECTED-TAVR^13^ recently reporting on 3000 randomized patients. Given these new data, we performed an updated systematic review and meta-analysis to investigate the effect of EPDs on clinical efficacy, safety, and neuroimaging parameters.

## Methods

This systematic review and meta-analysis was conducted according to PRISMA guidance.^14^ The study was registered a priori on the PROSPERO database (CRD42022377939).

### Search strategy

We performed a systematic search of the MEDLINE and Embase databases from inception through October 24, 2022, for RCTs comparing EPDs with control during TAVR. No filters or limits were applied. Search strings included (“transcatheter aortic valve implantation” OR “transcatheter aortic valve replacement”) AND (“embolic protection” OR “cerebral protection”). Hand search of included studies was used. All authors independently participated in searching and screening with disputes resolved by consensus.

### Inclusion and exclusion criteria

All randomized trials comparing EPD with controls were considered eligible for inclusion.

### End points

The primary end point was risk of all stroke. Secondary clinical end points included risk of death, disabling stroke, nondisabling stroke, all bleeding, life-threatening or disabling bleeding, vascular complications, and acute kidney injury.

Neuroimaging end points included total lesion volume (TLV) and number of ischemic magnetic resonance imaging (MRI) lesions. Outcomes with the longest follow-up were used. Definitions for individual end points provided by the included studies are summarized in Supplemental Table S1. Further details on diffusion-weighted MRI protocols used in the included studies and usage of imaging core laboratories are summarized in Supplemental Table S2.

### Data extraction and quality assessment

Three authors (R.K.R., Y.A., J.P.H.) independently participated in data extraction with data abstracted onto study-specific collection forms. Relevant study characteristics included first author, study acronyms, year of publication, study regions, number of patients, mean age, follow-up durations, inclusion and exclusion criteria, device type, TAVR type, and primary outcomes. Disputes were resolved by consensus discussion. The Cochrane risk-of-bias tool^15^ was applied to each included trial.

### Statistical analysis

Tests for publication bias were planned in the event of 10 or more studies being eligible for inclusion. For binary end points, we extracted event rates and pooled relative risks (RR). For primary analyses on neuroimaging end points, TLVs were extracted when studies reported values based on whole-brain MRI rather than those on protected-brain MRI because this was deemed more clinically relevant. For inclusion in sensitivity analysis, TLVs were extracted when studies additionally reported values based on protected-brain MRI. TLVs are presented as mean difference (MD) ± SD. If individual studies reported medians with corresponding interquartile range or 95% CI, these values were converted to mean ± SD using the methods described by Luo et al^16^ and Wan et al^17^ to facilitate quantitative synthesis.

Random-effects meta-analyses were performed using the restricted maximum likelihood estimator. All analyses were performed based on the intention-to-treat principle. The *I*^2^ statistic was used to assess heterogeneity.

Given that different EPDs are available, sensitivity analyses were performed to synthesize trials studying the same device to consider clinical heterogeneity caused by the device type. All analyses were performed within the statistical programming environment R using the metafor package. *P* values of <.05 were considered statistically significant.

## Results

**Figure 1:**
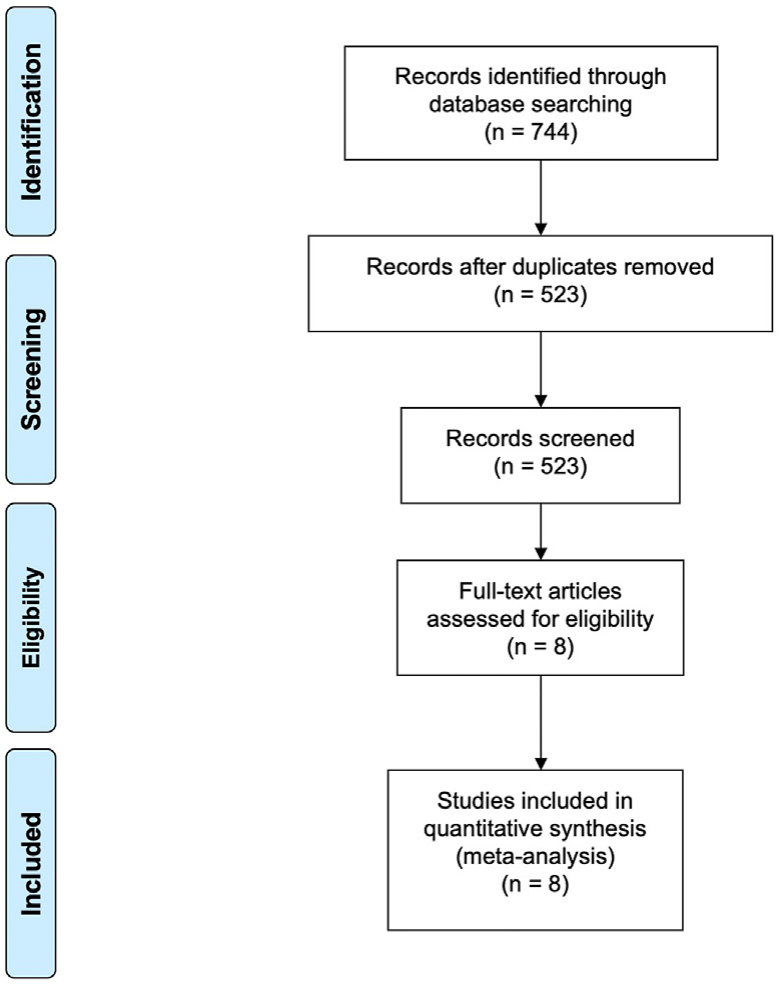
**Flowchart detailing the study selection process**.

Eight RCTs^11^, ^12^, ^13^^,^^18^, ^19^, ^20^, ^21^, ^22^ enrolling 4043 patients met the inclusion criteria, with 2175 randomly assigned to EPDs and 1868 to control. The following EPDs were studied: the Sentinel (Boston Scientific), the Montage (Claret Medical), the TriGUARD (Keystone Heart) and the EMBOL-X (Edwards Lifesciences). A flow chart representing the study selection process is displayed in Figure 1. Full study characteristics are displayed in Table 1. Because only 8 studies were included, tests for publication bias were not performed, as per Cochrane recommendations.^23^

**Table 1:** Characteristics of the included studies.

Study, year	Region	N	Mean age^a^	Follow-up	Entry criteria	Device	TAVR type	Primary outcome	Outcome definitions
PROTECTED-TAVR,^13^ 2022	USA, Europe, and Australia	3000	78.9 ± 8 y in the embolic protection group; 78.9 ± 7.8 y in the control group	72 h after TAVR or before discharge for all 30 d for population who experienced a stroke	Documented aortic valve stenosis and is planned for treatment with an approved transcatheter aortic valve replacement device through transfemoral access. Exclusion criteria: Arterial stenosis >70% in either the left common carotid artery or the brachiocephalic artery. The brachiocephalic or left carotid artery reveals significant stenosis, ectasia, dissection, or aneurysm at the aortic ostium or within 3 cm of the aortic ostium. Compromised blood flow to the right upper extremity. Access vessels with excessive tortuosity. Uncorrected bleeding disorders. Contraindicated for anticoagulant and antiplatelet therapy	Sentinel	Balloon-expandable or nonballoon-expandable device; 100% transfemoral	Clinical stroke within 72 h after TAVR or before discharge (whichever came first)	The primary endpoint of all stroke (hemorrhagic, ischemic, or undetermined status) through 72 h after TAVR procedure or discharge (whichever came first) was defined by Neurologic Academic Research Consortium (NeuroARC) definitions.^24^ Additional measurements based on NeuroARC^24^ and Valve Academic Research Consortium (VARC)^25^^,^^26^ endpoints and definitions were assessed through 72 h after TAVR procedure or discharge (whichever came first)
REFLECT I,^11^ 2021	USA and Europe	258	80.4 ± 7.5 y in the roll-in embolic protection group 79.8 ± 7.3 y in the randomized embolic protection group 81.5 ± 7.1 y in the control group	30 d for safety, efficacy and DW-MRI outcomes 90 d for safety and efficacy outcomes	Symptomatic severe aortic stenosis referred for TAVR Exclusion criteria: Recent (<72 h) acute myocardial infarction, recent (<6 mo) stroke or transient ischemic attack, cardiogenic shock, impaired renal function (glomerular filtration rate <30 mL/min/1.73 m^2^), a history of bleeding diathesis or coagulopathy or contraindications to antiplatelet or anticoagulant therapy, and previous prosthetic valve implantation (including planned aortic valve-in-valve procedure), known hypersensitivity to device component materials or contrast that could not be adequately premedicated, severe peripheral artery disease that precluded vascular access, a severely atheromatous aortic arch, contraindications to cerebral MRI, planned cardiac intervention during or within 10 days before TAVR or if treatment with any other investigational device or procedure was planned during study period.	TriGuard HDH	Balloon-expandable Edwards SAPIEN valves (Edwards Lifesciences) in 62.5% of patients with TG and 59.7% of controls whereas self-expanding CoreValve transcatheter valves (Medtronic) were used in 32.4% of patients with TG and 35.5% of control patients; the remaining 5% of subjects received other commercial valves; 100% transfemoral	The primary safety end point measured at 30 d was a composite of all-cause death, stroke, life-threatening or disabling bleeding, stages 2-3 AKI, coronary artery obstruction requiring intervention, major vascular complications and valve-related dysfunction requiring repeat procedure. The primary efficacy end point was a hierarchical composite of (1) all-cause mortality or any stroke at 30 d, (2) National Institutes of Health stroke score (NIHSS) worsening from baseline to 2-5 d postprocedure or MoCA worsening at 30 d, and (3) total volume of cerebral ischemic lesions	The primary safety end point measured at 30 d was defined according to VARC-2^25^ as a composite of all-cause death, stroke, life-threatening or disabling bleeding, stages 2-3 AKI, coronary artery obstruction requiring intervention, major vascular complications and valve-related dysfunction requiring repeat procedure. The primary efficacy end point was a hierarchical composite of (1) all-cause mortality or any stroke at 30 d, (2) NIHSS worsening from baseline to 2-5 d postprocedure or MoCA worsening (decrease of 3 points or more from baseline) at 30 d, and (3) total volume of cerebral ischemic lesions detected by DW-MRI performed 2-5 d postprocedure
REFLECT II,^12^ 2021	USA	220	81.32 ± 6.97 y in the roll-in embolic protection group 79.71 ± 7.96 y in the embolic protection group 78.05 ± 8.19 y in the control group	30 d	Symptomatic severe aortic stenosis referred for transfemoral TAVR **Exclusion criteria:** previously implanted prosthetic aortic valves, recent acute myocardial infarctions (<72 h), strokes, or transient ischemic attacks in the past 6 mo, cardiogenic shock, bleeding histories, or contraindications to antiplatelet or anticoagulation therapy. Other exclusion criteria were impaired renal function (estimated glomerular filtration rate <30 mL/min), hepatic failure, planned treatment with another investigational device or procedure during the study period, other cardiac intervention planned or performed within 10 d before the TAVR procedure, or contraindication to cerebral MRI.	TriGUARD 3	Approximately one-third of the patients received self-expanding CoreValve transcatheter valves (Medtronic), and two-thirds received balloon-expandable SAPIEN valves (Edwards Lifesciences); 100% transfemoral	The primary combined safety end point measured at 30 d was a composite of all-cause mortality, stroke, life-threatening or disabling bleeding, stage 2 or 3 acute kidney injury, coronary artery obstruction requiring intervention, major vascular complication, and valve-related dysfunction requiring intervention. The primary efficacy end point was a hierarchical composite determined by pairwise comparison between all patients of: (1) all-cause mortality or any stroke at 30 d; (2) worsening NIHSS score at 2-5 d; (3) freedom from any cerebral ischemic lesions at 2-5 d; and (4) total volume of cerebral ischemic lesions	The primary combined safety end point measured at 30 d was a VARC-2–defined^25^ composite of all-cause mortality, stroke, life-threatening or disabling bleeding, stage 2 or 3 acute kidney injury, coronary artery obstruction requiring intervention, major vascular complication, and valve-related dysfunction requiring intervention. The primary efficacy end point was a hierarchical composite determined by pairwise comparison between all patients of: (1) all-cause mortality or any stroke at 30 d; (2) worsening NIHSS score at 2-5 d; (3) freedom from any cerebral ischemic lesions detected on DW-MRI at 2-5 d; and (4) total volume of cerebral ischemic lesions detected on DW-MRI at 2-5 d
SENTINEL,^19^ 2017	USA and Germany	363	83.4 y (78.0-88.2)	30 d	Severe symptomatic aortic stenosis and planned TAVR who were at high surgical risk **Key exclusion criteria:** Contraindications for right radial or brachial artery access and inability to undergo MRI brain evaluation for any reason	Sentinel	Any approved valve (70.2% balloon-expandable)	**Primary safety:** Occurrence of MACCE at 30 d compared with a historical performance goal **Primary efficacy:** Reduction in median total new lesion volume in protected territories between the device and control arms	The MACCE composite endpoint constituents included: all death; all strokes (disabling and nondisabling, VARC-2^25^; and acute kidney injury (stage 3, VARC-2). Stroke occurrence was assessed by neurologist-administered NIHSS and modified Rankin score at baseline (<14 d pre-procedure), discharge, and 30 d. For patients experiencing a stroke within 30 d, 90-d NIHSS and modified Rankin score were also administered by a neurologist to determine stroke severity. The primary efficacy endpoint was assessed by diffusion-weighted MRI at 2 to 7 d after TAVR. Total new lesion volume was defined as the sum of all diffusion- positive new cerebral lesion volumes in post- procedural scans relative to the pre-TAVR scans
CLEAN-TAVI,^20^ 2016	Germany	100	80.0 ± 5.1 y in the embolic protection group; 79.1 ± 4.1 y in the control group	7 d	Symptomatic severe aortic stenosis **Key exclusion criteria:** Anatomy unsuitable for a safe TAVR, preexisting permanent pacemaker, stroke within the past 12 months, carotid artery stenosis of >70%, significant stenosis of the right subclavian artery or the brachiocephalic trunk	Montage	CoreValve	Numerical difference in new positive postprocedural DW-MRI brain lesions at 2 d after TAVR in potentially protected territories	Neurologic outcomes were assessed by serial neurologic and neurocognitive assessment (NIHSS, modified Rankin scale, Montreal cognitive assessment). Procedural outcomes were assessed with VARC-2^25^ definitions
MISTRAL-C,^18^ 2016	Europe	65	81 y (78-85)	30 d	Symptomatic severe aortic stenosis planned for transfemoral TAVR **Key exclusion criteria:** Presence of a permanent pacemaker or automated internal cardiac defibrillator at baseline, a history of stroke with sequelae and dementia	Sentinel	Any available valve (69% balloon-expandable)	New cerebral lesions by DW-MRI 5-7 d after TAVR	For neurologic outcomes, a trained neurology specialist performed a comprehensive neurological exam, including the NIHSS and the modified Rankin Scale, and a neurocognitive evaluation with the Montreal Cognitive Assessment and the Mini-Mental State Examination. VARC-2^25^ definitions were applied to report relevant clinical end- points
EMBOL-X,^21^ 2015	Germany	30	81.1 ± 5.0 y in the embolic protection group; 82.1 ± 4.1 y in the control group	7 d	Consecutive patients with severe aortic stenosis who underwent transaortic TAVR	EMBOL-X	Sapien-XT	Number and size of new ischemic cerebral lesions within 7 d after TAVR	Clinical outcome definitions were not provided
DEFLECT III,^22^ 2015	Europe and Israel	85	82.5 ± 6.5 y in the embolic protection group; 82.3 ± 6.0 y in the control group	30 d	Severe symptomatic aortic stenosis referred for TAVR **Key exclusion criteria:** Recent myocardial infarction, previous stroke, cardiogenic shock, contraindications to antiplatelet or anticoagulant therapy, heavily calcified or severely atheromatous aortic arch or aortic arch anatomy that could prevent positioning and stability of the device, showed contraindications to cerebral MRI	TriGuard HDH	Any available (63.5% balloon-expandable)	In-hospital MACCE	The MACCE composite endpoint constituents included: all-cause mortality, all stroke (disabling and non-disabling), life-threatening (or disabling) bleeding, acute kidney injury (stage 2 or 3), and major vascular complications. All endpoints were defined according to VARC-2 definitions.^25^

DW-MRI, diffusion-weighted magnetic resonance imaging; MACCE, major adverse cardiovascular and cerebrovascular events; MRI, magnetic resonance imaging; TAVR, transcatheter aortic valve replacement.

a Mean age ± SD given for overall population if provided; otherwise given for each group. If mean age not available, then median with IQR given.

### Risk-of-bias assessment

Overall study quality after risk-of-bias assessment was high for 6 studies^11^, ^12^, ^13^^,^^19^^,^^20^^,^^22^ and moderate for 2.^18^^,^^21^ The complete results of risk-of-bias assessment are displayed in Table 2.

**Table 2:** Risk-of-bias assessment.

Reference, year	Random sequence generation	Allocation concealment	Blinding of participants and personnel	Blinding of outcome assessment	Incomplete outcome data	Selective reporting	Overall Quality
Kapadia et al,^13^ 2022	Low risk	Low risk	Low risk	Low risk	Low risk	Low risk	High
Lansky et al,^11^ 2021	Low risk	Low risk	Low risk	Low risk	Low risk	Low risk	High
Nazif et al,^12^ 2021	Low risk	Low risk	Low risk	Low risk	Low risk	Low risk	High
Kapadia et al,^19^ 2017	Low risk	Low risk	Low risk	Low risk	High risk	Low risk	High
Haussig et al,^20^ 2016	Low risk	Low risk	Low risk	Low risk	Low risk	Low risk	High
Van Mieghem et al,^18^ 2016	Unclear	Unclear	Low risk	Low risk	Low risk	Low risk	Moderate
Wendt et al,^21^ 2015	Unclear	Unclear	Low risk	Unclear	Low risk	Low risk	Moderate
Lansky et al,^22^ 2015	Unclear	Low risk	Low risk	Low risk	Low risk	Low risk	High

### Clinical efficacy: all stroke, disabling stroke, nondisabling stroke, and all-cause mortality

**Figure 2:**
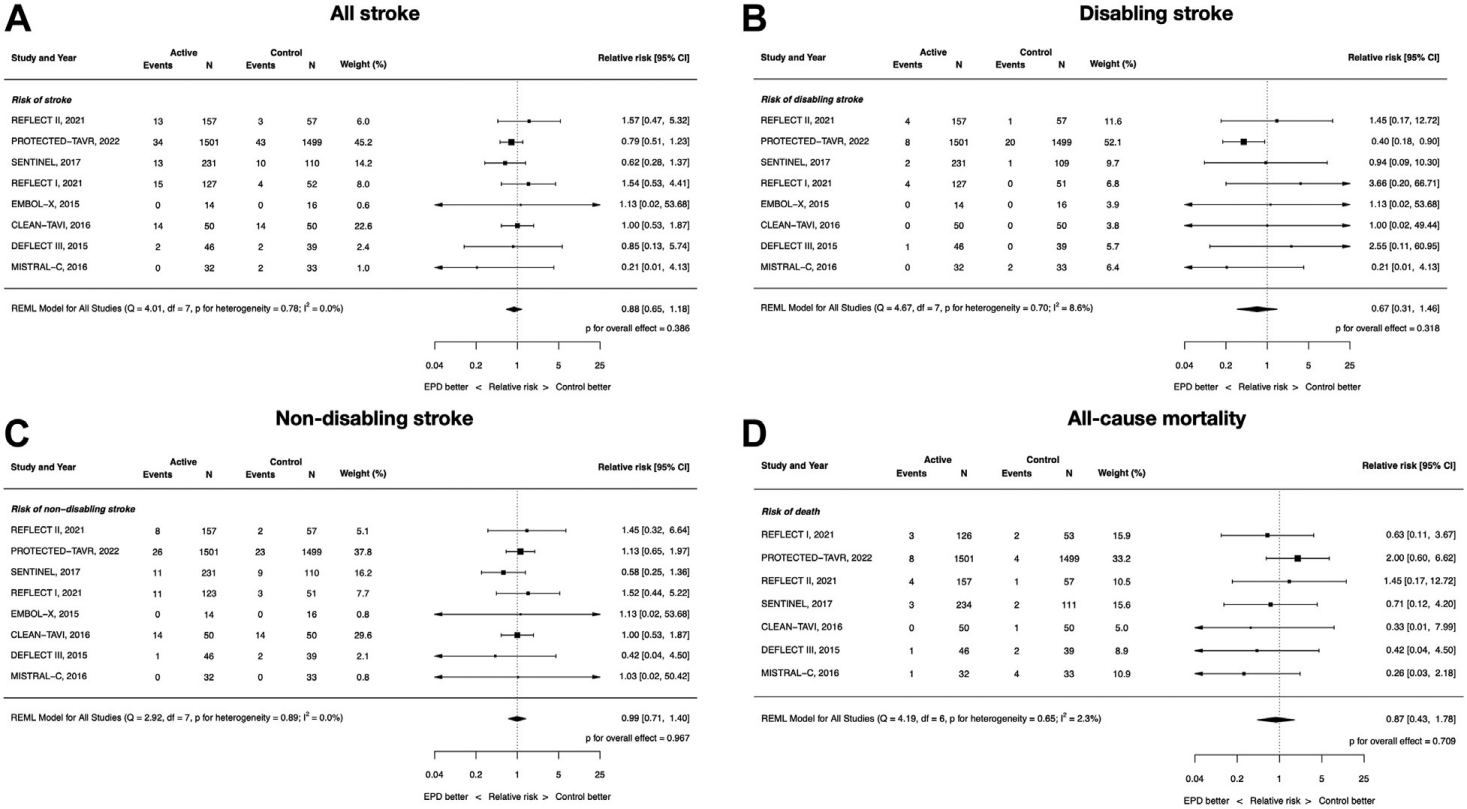
**Clinicalefficacy of cerebral embolic protection devices during transcatheter aortic valve replacement**. (**A**) All stroke; (**B**) disabling stroke; (**C**) nondisabling stroke; (**D**) all-cause mortality.

All 8 included RCTs contributed data to these end points except all-cause mortality because the EMBOL-X trial did not report mortality outcomes. The RR of all stroke with EPDs vs control was 0.88 (95% CI, 0.65-1.18; *P* = .39; *I*^2^ = 0%). There were no differences between EPDs and control in disabling (RR, 0.67; 95% CI, 0.31-1.46; *P* = .32; *I*^2^ = 8.6%) and nondisabling stroke (RR, 0.99; 95%, CI 0.71-1.40; *P* = .97; *I*^2^ = 0%). Similarly, there was no difference in all-cause mortality (RR, 0.87; 95% CI, 0.43-1.78; *P* = .71; *I*^2^ = 2.3%). (Figure 2).

### Clinical safety: bleeding, vascular complications, and acute kidney injury

**Figure 3:**
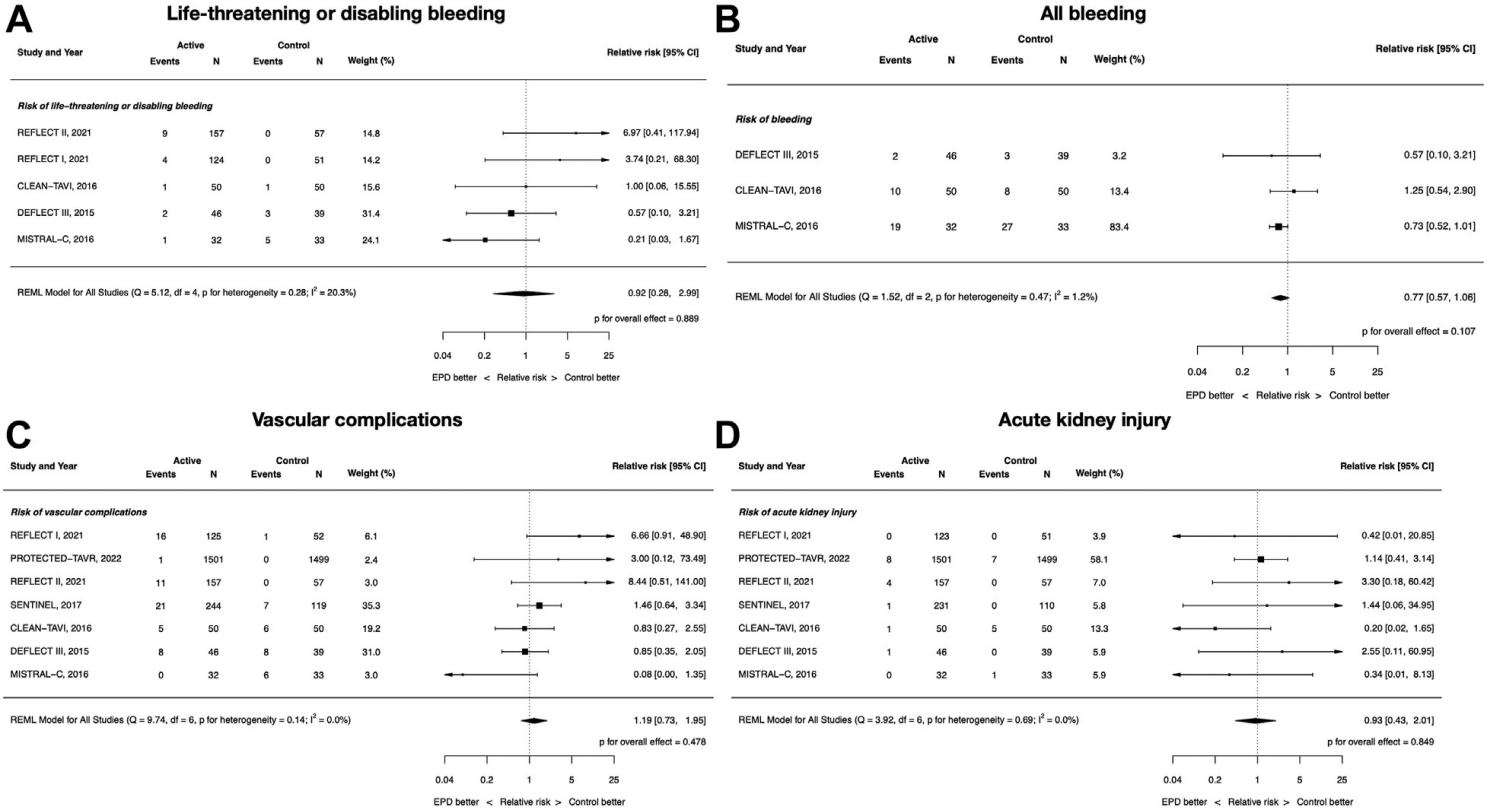
**Clinical safety of cerebral embolic protection devices during transcatheter aortic valve replacement.** (**A**) Life-threatening or disabling bleeding; (**B**) all bleeding; (**C**) vascular complications; (**D**) acute kidney injury.

There were no differences between EPDs and control in life-threatening or disabling bleeding (RR, 0.92; 95% CI, 0.28-2.99; *P* = .89; *I*^2^ = 20.3%), all bleeding (RR, 0.77; 95% CI, 0.57-1.06; *P* = .11; *I*^2^ = 1.2%), vascular complications (RR, 1.19; 95% CI, 0.73-1.95; *P* = .48; *I*^2^ = 0%), or acute kidney injury (RR, 0.93; 95% CI, 0.43-2.01; *P* = .85; *I*^2^ = 0%) (Figure 3).

### Neuroimaging parameters: TLV and the number of patients with new ischemic lesions

**Figure 4:**
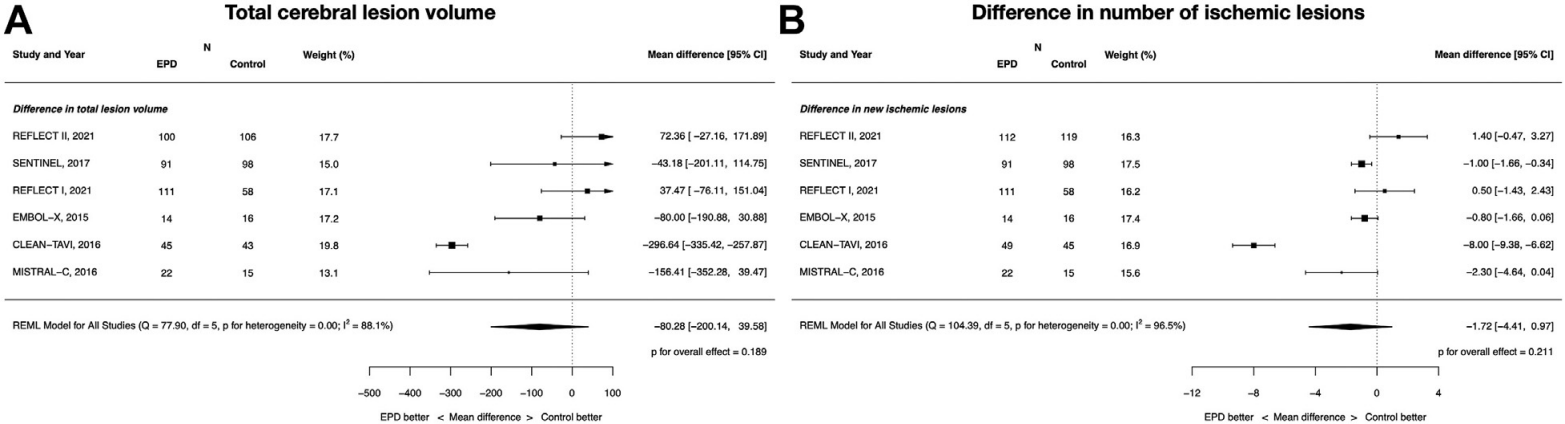
**Neuroimaging parameters of cerebral embolic protection devices during transcatheter aortic valve replacement.** (**A**) Total cerebral lesion volume in the whole brain; (**B**) difference in the number of ischemic lesions.

EPDs did not lead to reductions in whole-brain TLV (MD, −80.28 mm^3^; 95% CI, −200.14 to 39.58; *P* = .19; *I*^2^ = 88.1%) or the number of ischemic lesions (MD −1.72, 95% CI −4.41 to 0.97; *P* = .21; *I*^2^ = 96.5%). These data are displayed in Figure 4. EPDs resulted in a significant reduction in protected-brain TLV (MD, −92.74 mm^3^; 95% CI, −166.37 to −19.11; *P* = .014; *I*^2^ = 78.2%) (Supplemental Figure S1).

### Sensitivity analysis: clinical efficacy of the Sentinel device

**Figure 5:**
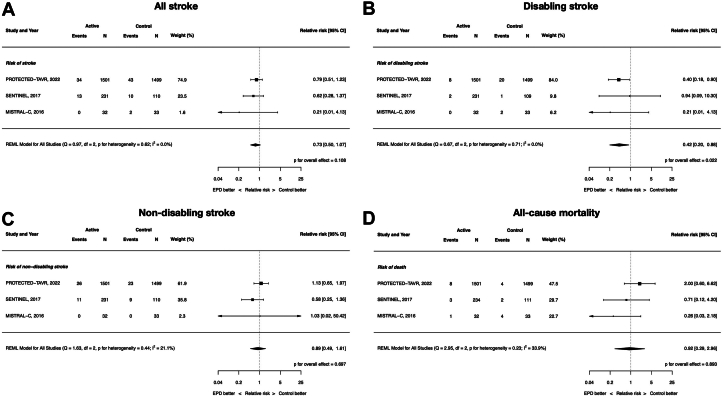
**Sensitivity analysis of clinical efficacy of the Sentinel cerebral embolic protection device during transcatheter aortic valve replacement.** (**A**) All stroke; (**B**) disabling stroke; (**C**) nondisabling stroke; (**D**) all-cause mortality.

Given that 3506 of the 4043 patients in this meta-analysis were randomly assigned in the studies of the Sentinel device, a meta-analysis of the MISTRAL-C,^18^ SENTINEL,^19^ and PROTECTED-TAVR^13^ trials was performed to parse the role of this specific device on clinical efficacy outcomes. The RR of all stroke with the Sentinel EPD vs control was 0.73 (95% CI, 0.50-1.07; *P* = .11; *I*^2^ = 0%). The Sentinel EPD reduced the risk of disabling stroke vs control (RR, 0.42; 95% CI, 0.20-0.88; *P* = .022; *I*^2^ = 0%) but did not significantly alter rates of nondisabling stroke (RR, 0.89; 95% CI, 0.49-1.61; *P* = .70; *I*^2^ = 0%). Similarly, there was no difference in all-cause mortality (RR, 0.92; 95% CI, 0.29-2.96; *P* = .90; *I*^2^ = 33.9%) (Figure 5).

## Discussion

This study synthesized all available randomized data for EPDs in patients who underwent TAVR, including 4043 patients (Central Illustration). When all trials were pooled, there were no differences in the rates of stroke, mortality, safety, or neuroimaging end points with EPDs compared with those of controls. In sensitivity analyses focusing solely on trials evaluating the Sentinel EPD (which is the device used in clinical practice today), a significant reduction in disabling stroke of ∼60% was seen. There were no signals of harm detected with EPD, confirming a favorable safety profile.

**Central Illustration:**
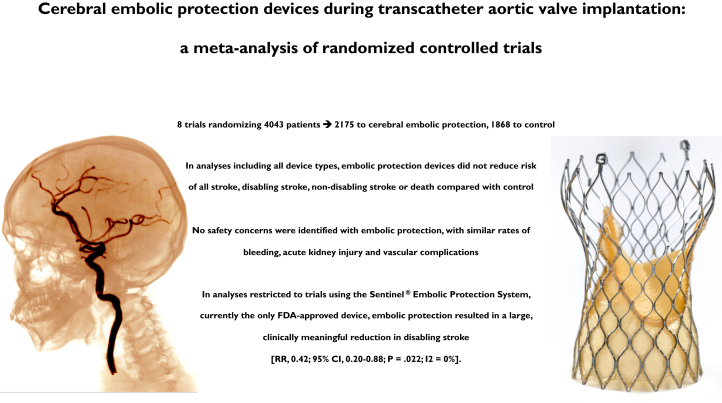
Cerebral embolic protection devices during transcatheter aortic valve replacement: a meta-analysis of randomized controlled trials. FDA, Food and Drug Administration; RR, relative risk.

Stroke rates in the decade since the inception of TAVR have remained >2% despite advancements in understanding, operative technique, and technology.^8^ Approximately half of these occur immediately or a few hours after TAVR^23^ and are mostly attributed to intraprocedural embolism. In addition to clinically evident strokes, TAVR causes “silent” brain lesions,^27^^,^^28^ relevant for their potential association with subtle functional and neurocognitive deficits that may go unnoticed. Furthermore, silent infarcts more than double the risk of subsequent stroke and dementia.^29^ Therefore, there has been interest in the potential role of EPDs.

This study demonstrated that when all RCTs investigating EPDs were pooled, there were no differences in stroke, whether disabling or nondisabling, and all-cause mortality. Similarly, there were no safety concerns identified with EPD. These findings have been reported in previous, smaller meta-analyses of solely RCTs.^10^^,^^30^^,^^31^ This may be due to low event rates across the trials for the relatively rare end points of death and stroke, which the meta-analysis technique helps to overcome through quantitative synthesis. However, this meta-analysis still likely remains underpowered for these low-frequency clinical events. Moreover, differences in intraprocedural heparinization use may have affected trial results, salient because EPD implantation results in longer procedural duration, greater device manipulation within the aortic arch, and increased thrombogenic surfaces. Thus, particular care is required surrounding intraprocedural anticoagulation. If not accounted for, the additional upfront embolic risk presented by EPDs may paradoxically increase stroke rates, precluding any benefit and biasing toward the null. Similarly, differing postprocedural anticoagulation and antiplatelet strategies or adherence may have resulted in a bias across trials. There were also no differences in whole-brain neuroimaging end points, with high heterogeneity demonstrated in these analyses. Reasons for this may include differences in measuring or reporting ischemic lesions and TLV, the timing of acquisition and imaging systems used. However, there was a reduction in protected-brain TLV, which is plausible given that EPDs can only prevent embolic debris to anatomical regions they shield. Achieving complete cerebral coverage should be a priority for future device iterations.

Importantly, this study highlights that use of the Sentinel EPD results in a large, statistically significant and clinically meaningful reduction in disabling stroke. This analysis is perhaps the most clinically relevant to TAVR operators in the United States, given that the Sentinel remains the only Food and Drug Administration approved device available for general use. Despite the relative rarity of stroke as a complication, the devastating consequences and possibility of occurrence in healthy patients with low-risk TAVR patients warrants attention. A recent meta-analysis demonstrated that early stroke risk is lower in low-risk TAVR vs surgical aortic valve replacement, although this difference was not significant 1-year postprocedure and is still nonzero.^32^ The finding that the Sentinel device did not reduce all-cause mortality may be considered surprising given that stroke incidences after TAVR are independently associated with markedly worse 30-day cardiovascular and all-cause mortality.^33^ However, this analysis was underpowered for mortality. Furthermore, nonfatal but disabling stroke may lead to a longer-term increase in mortality, which is not captured in the randomized trials with short-term follow-up. Similarly, the risk of nondisabling stroke was not reduced, which is biologically plausible given the Sentinel’s mesh pore size of 140 μm may not catch all embolized particulate matter smaller than this. In addition, the Sentinel device protects the brachiocephalic trunk and left common carotid artery but not the left subclavian artery, representing incomplete cerebral coverage.

There are a few reasons why the analysis focusing on the Sentinel device may have demonstrated significantly reduced disabling strokes compared with the overall pooled result. Practically, the Sentinel requires a smaller 6F delivery sheath compared with the 9F TriGuard and 17F EMBOL-X filter sheaths, presumably resulting in less trauma to both vessel and valve during aortic arch navigation. Mechanistically, the Sentinel is distinct in that it captures particulate debris and facilitates retrieval rather than relying on deflection as with other EPDs. Most importantly, the Sentinel trial is the best-studied device owing to the PROTECTED-TAVR^13^ trial with 3000 patients. The trials of other devices were likely too small to suggest any effect on clinical events.

Unlike previous smaller meta-analyses, we investigated individual clinical end points rather than pooling data and generating composites, negating the risk of counting events twice when trials provide time-to-event data. Statistical heterogeneity was mostly low. We considered clinical heterogeneity posed by differing device types by performing sensitivity analyses including only trials evaluating the Sentinel device, which contributed ∼90% of patients to the overall pooled analysis.

This study-level meta-analysis is naturally subject to the inherent limitations of the constituent RCTs, which include but are not limited to low clinical event rates, treatment crossover, and loss-to-follow-up, particularly for neurologic assessment and imaging end points. Owing to the use of aggregate data, hypothesis-generating subgroup analyses based on prerandomization demographic characteristics were inappropriate. An individual participant data meta-analysis is planned to combine PROTECTED-TAVR and the ongoing British Heart Foundation-funded PROTECT-TAVI (ISRCTN16665769). PROTECT-TAVI aims to enroll nearly 8000 randomized patients by 2026 and will define further the role of the Sentinel EPD. Subgroup analyses may identify higher-risk patients deriving benefit from EPDs, such as those with heavily calcified aortic anatomy or bicuspid aortic valves. Furthermore, in observational analyses, independent baseline predictors of periprocedural stroke include age older than 85 years, body mass index of <25 kg/m^2^, and a history of coronary artery disease, whereas previous cerebrovascular events and an estimated glomerular filtration rate of <30 mL/min/1.73 m^2^ independently associate with 30-day stroke risk.^34^ These relevant subgroups should be further interrogated. Finally, future EPD iterations might improve efficacy by permitting complete cerebral coverage. Until then, this study represents the best available evidence within the field.

In conclusion, in this analysis of all 8 published RCTs evaluating all EPDs in TAVR, there was no evidence for differences in rates of stroke, mortality, or safety outcomes compared with controls. Importantly, in analyses limited to the Sentinel trials, ∼60% reduction in disabling stroke was demonstrated. However, equipoise remains, and efforts should focus on enrolment into ongoing RCTs alongside development of newer devices enabling complete cerebral coverage.
