# Supplementary material for: Cerebral Embolic Protection Devices During Transcatheter Aortic Valve Replacement: A Meta-analysis of Randomized Controlled Trials
Source: J Soc Cardiovasc Angiogr Interv. 2023 Aug 25;2(5):101031. doi: 10.1016/j.jscai.2023.101031 (PMC10533415; doi:10.1016/j.jscai.2023.101031)
Supplement: Supplemental Tables and Supplemental Figure S1 [file mmc1.docx]

**Supplementary Material**

**Cerebral embolic protection devices during transcatheter aortic valve implantation: a meta-analysis of randomized controlled trials.**

Rohin K Reddy MBBS BSc^1^, Yousif Ahmad BMBS PhD^2^, Ahran D Arnold MBBS PhD^1^, James P Howard BM BChir PhD^1^

^1^*National Heart and Lung Institute, Imperial College London, London, UK*

^2^*Section of Cardiovascular Medicine, Yale University, New Haven, Connecticut, USA*

**Contents**

**Table S1. Outcome definitions used in individual trials**

**Table S2. Details of neuroimaging protocols used in individual trials**

**Figure S1. Effects of cerebral embolic protection devices during transcatheter aortic valve implantation on total cerebral lesion volume in the protected brain**

**Table S1. Outcome definitions used in individual trials**

| **Author** | **Study acronym** | **Outcome definitions** | **Outcome adjudication** |
| --- | --- | --- | --- |
| Kapadia *et al.* | PROTECTED-TAVR | **Stroke**  Defined as an acute episode of a focal or global neurologic dysfunction caused by vascular injury to the brain, spinal cord, or retina resulting from hemorrhage or infarctions. A neurology professional (board-certified or board-eligible neurologist, neurology fellow, neurology physician assistant, or neurology nurse practitioner) performed neurologic examinations at baseline (after randomization) and after TAVI. In patients in whom stroke was suspected, neuro- imaging was performed according to the standard of care at each site at the discretion of the treating physician. Routine neuroimaging was not performed to identify covert (asymptomatic) brain infarction. An independent clinical events committee adjudicated all stroke events, with stroke subtype characterized according to definitions of the Neurologic Academic Research Consortium (NeuroARC): type 1.a to 1.d and type 2.b events were considered to be strokes.  **AKI**  Based on the AKIN System Stage 3 (including renal replacement therapy) and Stage 2.  **Vascular access related complications**  Defined as Sentinel access site vascular complications related to the procedure (major and minor). | Mortality (cardiovascular and non- cardiovascular), neurological endpoints (stroke, transient ischemic attack [TIA], and delirium), acute kidney injury, and CEP access site major vascular complications were adjudicated by an independent CEC. |
| Lansky *et al.* | REFLECT I | All-cause death, stroke, life-threatening or disabling bleeding, acute kidney injury (AKI) stage 2–3, major vascular complications were all defined according to the Valve Academic Research Consortium 2 (VARC-2). | An independent Clinical Events Committee (CEC) adjudicated all potential endpoint events. |
| Nazif *et al.* | REFLECT II | All-cause death, stroke, life-threatening or disabling bleeding, acute kidney injury (AKI) stage 2–3, major vascular complications were all defined according to the Valve Academic Research Consortium 2 (VARC-2). | An independent clinical events committee adjudicated all potential endpoint events. |
| Kapadia *et al.* | SENTINEL | MACCE was defined as follows: all death; all strokes (disabling and nondisabling, VARC-2 [Valve Academic Research Consortium-2]); and acute kidney injury (stage 3, VARC-2). | No information provided. |
| Haussig *et al.* | CLEAN-TAVI | Death, stroke, TIA, or bleeding were assessed the definitions established by the Valve Academic Research Consortium-2. Vascular complications were defined as any aortic dissection, aortic rupture, annulus rupture, left ventricle perforation, OR new apical aneurysm/pseudo-aneurysm OR access site or access-related vascular injury (dissection, stenosis, perforation, rupture, arterio- venous fistula, pseudoaneurysm, hematoma, irreversible nerve injury, compartment syndrome, percutaneous closure device failure) leading to death, life-threatening or major bleeding, visceral ischemia, or neurological impairment OR distal embolization (non-cerebral) from a vascular source requiring surgery or resulting in amputation or irreversible end-organ damage OR the use of unplanned endovascular or surgical intervention associated with death, major bleeding, visceral ischemia or neurological impairment OR any new ipsilateral lower extremity ischemia documented by patient symptoms, physical exam, and/or decreased or absent blood flow on lower extremity angiogram OR surgery for access site-related nerve injury OR permanent access site-related nerve injury. | No information provided. |
| Van Mieghem *et al.* | MISTRAL-C | The Valve Academic Research Consortium-2 definitions were applied to report relevant clinical endpoints. | No information provided. |
| Wendt *et al.* | EMBOL-X | No information provided on endpoint definitions. | No information provided. |
| Lansky *et al.* | DEFLECT III | All endpoints were defined according to Valve Academic Research Consortium-2. | All adverse events were adjudicated by an independent Clinical Events Committee (Yale Cardiovascular Research Group, New Haven, CT, USA), which included a cardiac surgeon, an interventional cardiologist, and a vascular neurologist. |

**Table S2. Details of neuroimaging protocols used in individual trials**

| **Author** | **Study acronym** | **Neuroimaging endpoints** |
| --- | --- | --- |
| Kapadia *et al.* | PROTECTED-TAVR | Not reported |
| Lansky *et al.* | REFLECT I | Brain DW-MRI using a standardized image acquisition protocol (1.5 T) was performed 2–5 days post-procedure to define the ischaemic burden of the TAVI procedure by an independent MRI Core Laboratory [Buffalo Neuroimaging Center, Buffalo, NY (BNAC)] using validated methods.  **DWI Acquisition**  DWI were acquired with a 2D echo planar sequence with one b=0 image and 3 orthogonal diffusion directions with b=1000 s/mm2. Other sequence parameters were not fully standardized across sites and values ranged [minimum – maximum]: repetition time (TR) = [2900 ms – 10800 ms], echo time (TE) = [60 ms – 145 ms], slice thickness = [ 3 mm – 5 mm (with 0mm to 2 mm interslice gap)] and in-plane resolution = [0.9 mm2 – 1.8 mm2]. In addition, fluid attenuated inversion recovery (FLAIR) images were acquired with a 2D spin echo inversion recovery sequence. FLAIR sequence parameters were not fully standardized across sites and values ranged [minimum – maximum]: TR = [4800 ms – 11000 ms], TE = [82 ms – 149 ms], inversion time (TI) = [1746 ms – 2800 ms]; slice thickness = [3 mm – 5 mm (with 0 mm to 2 mm interslice gap)] and in-plane resolution = [0.7 mm2 – 1.0 mm2].  Site-specific deviations in acquisition protocols were allowed to accommodate individual scanner capabilities, provided they were acquired consistently within the site.  **Analysis Methodology**  The diffusion b=0 (b0) and 3 orthogonal diffusion directions b=1000 diffusion-encoded raw images were combined to create trace and apparent diffusivity coefficient (ADC) images.  To facilitate direct analysis, all within-subject scans were co-registered to each subject’s FLAIR image using FMRIB’s Linear Image Registration Tool (FLIRT) with 6 degrees of freedom.(32) Corrected FLAIR and DWI trace images were standardized by applying a piecewise-linear histogram adjustment method to compensate for scan-to-scan variability in absolute intensity.  Lesions were delineated on corrected and aligned DWI trace images and FLAIR images using a semi-automated contouring technique provided by the Java Image Manipulation (JIM) software package, with simultaneous reference to the ADC and FLAIR images (Online Figure 1-1). Using this approach, a trained operator identified lesions individually, and for each lesion an assistive algorithm delineated a highly reproducible iso-contour at the maximum local gradient. The operator viewed all images simultaneously to increase confidence. FLAIR lesions were restricted to those that were not simultaneously DWI-positive, in order to quantify a proxy for pre-procedural lesion burden on post-procedural images.  Lesions were delineated on corrected and aligned DWI and FLAIR images using a semi-automated contouring technique, with simultaneous reference to the ADC images. In red are contoured ROIs, indicative of new ischemic lesions (left and center) and pre-existing FLAIR lesions (right). Note the removed FLAIR ROI on the upper right (patient left) due to simultaneous DWI-positivity. Legend: FLAIR - fluid attenuated inversion recovery; DWI - diffusion-weighted image; ADC - apparent diffusivity coefficient; ROI - region of interest.  In addition to lesion counts and volumetry, anatomical regions and vascular territory were assessed using an atlas-based technique. For this purpose, a vascular territory atlas was manually created in the standard MNI 152 template space(36) based on existing literature, including 28 separate regions. Individual FLAIR images were used to non-linearly align this atlas to individual lesion maps. First, individuals’ FLAIR images were corrected for intensity inhomogeneity using N3, then aligned to a FLAIR atlas in MNI 152 space using a two-stage process consisting of an initial rigid-body co-registration followed by composition with a warp field obtained from a non-linear warping technique. These transforms were then inverted, and applied to the original atlas. Lesions were assigned to discrete regions/territories based on the location of their 3-dimensional centroid. All stages were manually reviewed and corrected as necessary. Lesion number and volume within each anatomical and vascular territory were than assessed separately and used in clinical endpoint and event (CEC) adjudications.  The following post-procedural MRI efficacy endpoints of the study were calculated as planned: number of cerebral ischemic lesions, total volume of cerebral ischemic lesions, per-patient average single cerebral ischemic lesion volume, per-patient maximum cerebral ischemic lesion volume, per-lesion single cerebral ischemic lesion volume , per-patient non-DWI T2-FLAIR lesion volume (quantified as a proxy for baseline T2-FLAIR lesion volume).  In addition to subject-level statistical analysis exploratory and statistical visualization techniques were performed to more fully understand the dynamics of the device efficacy (BNAC, Buffalo Neuroimaging Analysis Center, Buffalo, NY, US). These post-hoc analyses were unblinded to treatment group assignments and clinical outcomes, but with unmodified individual lesion ROIs that were created during the blinded phase of analysis for each subject.  For lesion visualization, individual DW images and their associated lesion maps were non-linearly aligned (warped) to the standard MNI 152 template space. Visualizations were created by combining all lesion maps from all subjects into a single image for each group, using maximum intensity projection across the subject axis. Therefore, individual figures show the topographical and size distribution of all lesions from all subjects in each group. 3D Rendering of maps was created using the MayaVi scientific data visualization system.  Because the TriGuard device may protect lesions at a certain size/volume threshold, a comprehensive multi-threshold exploratory analysis was conducted looking at group differentiation as a function of lesion volume cut-off threshold ranging from 0 to 1000 mm3 in 100 mm3 increments. At each threshold, individual subjects’ supra-threshold cerebral ischemic lesion (SCIL) volume was retained to calculate SCIL volume. For each subject and for each threshold in turn, their SCIL volume and number above a given individual lesion cut-off are presented. Overall difference in slopes between groups was assessed via linear mixed effects modeling with cube-root transformed SCIL volume as the dependent variable, treatment, threshold, and treatment by threshold interaction as fixed effects, and subject-specific intercepts as random effects. Post-hoc per-threshold differences were assessed via T-tests. |
| Nazif *et al.* | REFLECT II | DW-MRI was performed using a standardized acquisition protocol (1.5 T) 2 to 5 days after TAVR, and images were analyzed in an independent MRI core laboratory (Buffalo Neuroimaging Center, Buffalo, New York) using validated methods.  Diffusion weighted images (DWI) were acquired with a 2D echo planar sequence with one b=0 image and 3 orthogonal diffusion directions with b=1000 s/mm2. Other sequence parameters were not fully standardized across sites and values are presented as ranges [minimum – maximum]: repetition time (TR) = [2900ms – 10800ms], echo time (TE) = [60ms – 145ms], slice thickness = [ 3mm – 5mm (with 0mm to 2 mm interslice gap)] and in-plane resolution = [0.9mm2 – 1.8mm2]. In addition, fluid attenuated inversion recovery (FLAIR) images were acquired with a 2D spin echo inversion recovery sequence. FLAIR sequence parameters were not fully standardized across sites and values are presented as ranges [minimum – maximum]: TR = [4800ms – 11000ms], TE = [82ms – 149ms], inversion time (TI) = [1746ms – 2800ms]; slice thickness = [3mm – 5mm (with 0mm to 2mm interslice gap)] and in-plane resolution = [0.7 mm2 – 1.0mm2].  Site-specific deviations in acquisition protocols were allowed to accommodate individual scanner capabilities, provided they were acquired consistently within the site.  The diffusion b=0 (b0) and 3 orthogonal diffusion directions b=1000 diffusion-encoded raw images were combined to create trace and apparent diffusivity coefficient (ADC) images.  To facilitate direct analysis, all within-subject scans were co-registered to each subject’s FLAIR image using FMRIB’s Linear Image Registration Tool (FLIRT) with 6 degrees of freedom.1 Corrected FLAIR and DWI trace images were standardized by applying a piecewise-linear histogram adjustment method to compensate for scan-to-scan variability in absolute intensity.2  Lesions were delineated on corrected and aligned DWI trace images and FLAIR images using a semi-automated contouring technique provided by the Java Image Manipulation (JIM) software package, with simultaneous reference to the ADC and FLAIR images. Using this approach, a trained operator identified lesions individually, and for each lesion an assistive algorithm delineated a highly reproducible iso-contour at the maximum local gradient. The operator viewed all images simultaneously to increase confidence. FLAIR lesions were restricted to those that were not simultaneously DWI-positive, in order to quantify a proxy for pre-procedural lesion burden on post-procedural images.  In addition to lesion counts and volumetry, anatomical regions and vascular territory were assessed using an atlas-based technique. For this purpose, a vascular territory atlas was manually created in the standard MNI 152 template space5 based on existing literature, including 28 separate regions. Individual FLAIR images were used to non-linearly align this atlas to individual lesion maps. First, individuals’ FLAIR images were corrected for intensity inhomogeneity using N3, then aligned to a FLAIR atlas in MNI 152 space using a two-stage process consisting of an initial rigid-body co-registration followed by composition with a warp field obtained from a non-linear warping technique.7 These transforms were then inverted, and applied to the original atlas. Lesions were assigned to discrete regions/territories based on the location of their 3-dimensional centroid. All stages were manually reviewed and corrected as necessary. Lesion number and volume within each anatomical and vascular territory were than assessed separately and used in clinical endpoint and event (CEC) adjudications.  The following post-procedural MRI efficacy endpoints of the study were calculated as planned: number of cerebral ischemic lesions, total volume of cerebral ischemic lesions, per-patient average single cerebral ischemic lesion volume, per-patient maximum cerebral ischemic lesion volume, per-lesion single cerebral ischemic lesion volume , per-patient non-DWI T2-FLAIR lesion volume (quantified as a proxy for baseline T2-FLAIR lesion volume).  In addition to subject-level statistical analysis exploratory and statistical visualization techniques were performed to more fully understand the dynamics of the device efficacy. These post-hoc analyses were unblinded to treatment group assignments and clinical outcomes, but with unmodified individual lesion ROIs that were created during the blinded phase of analysis for each subject.  For lesion visualization, individual DW images and their associated lesion maps were non-linearly aligned (warped) to the standard MNI 152 template space.5 Visualizations were created by combining all lesion maps from all subjects into a single image for each group, using maximum intensity projection across the subject axis. Therefore, individual figures show the topographical and size distribution of all lesions from all subjects in each group. 3D Rendering of maps was created using the MayaVi scientific data visualization system.8  Because the TriGUARD 3 device may protect lesions at a certain size/volume threshold, the data was also analyzed at different thresholds. Therefore, an additional comprehensive multi-threshold exploratory analysis was conducted looking at group differentiation as a function of lesion volume cut-off threshold ranging from 0 to 1000mm3 in 100mm3 increments. At each threshold, individual subjects’ total new supra-threshold cerebral ischemic lesion (SCIL) volume was retained. For each subject and for each threshold in turn, their SCIL volume and number above a given individual lesion cut-off are presented. Overall difference in slopes between groups was assessed via linear mixed effects modeling with cube-root transformed SCIL volume as the dependent variable, treatment, threshold, and treatment by threshold interaction as fixed effects, and subject-specific intercepts as random effects. Post-hoc per-threshold differences were assessed via t-tests. |
| Kapadia *et al.* | SENTINEL | Brain MRI using a 3-T scanner was performed in both imaging arms (device and control) at baseline and post-TAVR at 2 to 7 days and at 30 days. All MRI studies were analyzed by a core laboratory in a blinded manner.  MR images at each site were acquired only on a 3 Tesla certified and validated system at 0, 2-7 and 30 days. Diffusion weighted images (DWI) were acquired with a 2D echo planar sequence with one b=0 image and 3 orthogonal diffusion directions with b=1000 s/mm.1 Additional parameters were: repetition time (TR) = 13000ms, echo time (TE) = 100ms, slice thickness = 3mm (no gap), acquisition matrix 204 x 156, final voxel size = 1.25mm x 1.25mm x 3.0mm. The DWI images were required at baseline and 2-7 days post procedure on all evaluable imaging cohort patients. Fluid attenuated inversion recovery (FLAIR) images were acquired with a 2D spin echo inversion recovery sequence with an inversion time (TI) of 2580ms. Additional parameters were: TR = 9730ms, TE=92ms, slice thickness = 2mm (no gap), acquisition matrix 256 x 186, final voxel size = 0.94mm x 1.17mm x 2.0mm. The FLAIR images were required at baseline and 30 days post procedure on all evaluable imaging cohort patients. High resolution T1-weighted images (hires-T1) were acquired with an MP-RAGE sequence. Additional parameters were: TR = 1690ms, TE=2.57ms, flip angle (FA) = 12, TI=1100ms, slice thickness = 1.5mm (no gap), acquisition matrix 256 x 224, final voxel size = 1.00mm x 1.00mm x 1.5mm. Finally, either a manufacturer-based dual-echo GRE sequence was used to acquire B0 field maps (voxel size = 4.00mm x 4.00mm x 5.00mm), or the DWI images were acquired with two different phase encoding directions. Minor site-specific deviations were allowed to accommodate individual scanner capabilities, provided they were approved by MR physicists at the reading center and were acquired consistently within the site.  DWI acquisitions are subject to substantial artifacts, including eddy current distortions, susceptibility-induced warping, and signal dropout. Although these do not have a substantial impact on clinical assessment of large lesions associated with stroke or transient ischemic attack (TIA), they are quite large relative to the small embolic lesions resulting from the TAVI procedure – distortions may easily be on the order of 1cm, while lesions may be as small as a few mm. Therefore, a number of pre-processing steps were taken to improve image quality and subsequent analysis. First, the raw DWI images were corrected for distortions using FMRIB’s FSL FDT library. This was accomplished using either directly acquired fieldmaps or by inferring the field map from paired, phase-reversed DWI acquisitions.3 Next, the diffusion b=0 (b0) and three corrected b=1000 diffusion-encoded raw images were combined to create trace and apparent diffusivity coefficient (ADC) images.  Because the lesions are often so small, subtraction imaging was also employed to increase lesion salience. Baseline DWI and FLAIR images were voxel-wise subtracted from follow-up images to produce direct change maps. To facilitate this subtraction approach, additional pre-processing steps were performed. First, low-frequency spatial intensity inhomogeneities on FLAIR images were corrected using N3.5 Corrected FLAIR and DWI trace images were further standardized by applying a piecewise-linear histogram adjustment method to compensate for scan-to- scan variability in absolute intensity. Finally, to facilitate direct longitudinal analysis, all within-subject scans were co-registered to each subject’s baseline FLAIR image using FLIRT with 6 degrees of freedom.  Lesions were delineated on corrected and aligned 2-7, and 30 day DWI trace images and 30 day FLAIR images using a semi-automated contouring technique provided by the JIM software package, with simultaneous reference to the ADC and subtraction images. Using this approach, a trained operator identified lesions individually, and for each lesion an assistive algorithm delineated a highly reproducible iso-contour at the maximum local gradient. The operator viewed all images and change maps simultaneously to increase confidence and also coded lesions as new or persistent.  In addition to lesion counts and volumetry, vascular territory was also assessed using an atlas-based technique. For this purpose, a vascular territory atlas was manually created in the standard MNI 152 template space9 based on existing literature, and including 28 separate regions. Individual hires-T1 images were used to non-linearly align this atlas to individual lesion maps. First, individuals’ hires-T1 images were corrected for intensity inhomogeneity using N3, then aligned to the MNI 152 template using a two-stage process consisting of an initial rigid-body co- registration followed by composition with a warp field obtained from a non-linear warping technique. These transforms were then inverted, and applied to the original atlas. Lesion number and volume within each vascular territory were than assessed separately. |
| Haussig *et al.* | CLEAN-TAVI | MRI scans were analyzed in blinded fashion by the MRI core laboratory (Buffalo Neuroimaging Analysis Cen- ter, Buffalo, NY). The MRI protocol included diffusion- weighted images (DWIs) acquired with a 2D-echo planar sequence, high-resolution T1-weighted images acquired with an MP-RAGE sequence, and B0 field maps acquired with a manufacturer-based dual-echo gradient echo sequence. All examinations were acquired on a 3T scanner (Magnetom Verio) except for 11 patients who were pacemaker dependent following TAVI. For these patients, a 1.5T system (Intera by Philips) was used. MRI outcomes included calculation of number and volume of new DWIs (2 and 7 days) by subtraction of the existing baseline lesions in the whole brain and within predefined vascular territories (ie, the potentially protected and partially protected areas). None of the patients had any endovascular diagnostic tests or treatments performed between the baseline MRI and the TAVI.  For the majority of subjects, MR images were acquired on a 3 Tesla Siemens Verio system at 0, 2, 7 days. Diffusion weighted images (DWI) were acquired with a 2D echo planar sequence with 3 orthogonal diffusion directions with b values of both 500 and 1000 s/mm2. Additional parameters were: repetition time (TR) = 13000ms, echo time (TE) = 100ms, slice thickness = 3mm (no gap), acquisition matrix 204 x 156, final voxel size = 1.25mm x 1.25mm x 3.0mm. Fluid attenuated inversion recovery (FLAIR) images were acquired with a 2D spin echo inversion recovery sequence with an inversion time (TI) of 2580ms. Additional parameters were: TR = 9730ms, TE=92ms, slice thickness = 2mm (no gap), acquisition matrix 256 x 186, final voxel size = 0.94mm x 1.17mm x 2.0mm. High resolution T1-weighted images (hires-T1) were acquired with an MP-RAGE sequence. Additional parameters were: TR = 1690ms, TE=2.57ms, flip angle (FA) = 12, TI=1100ms, slice thickness = 1.5mm (no gap), acquisition matrix 256 x 224, final voxel size = 1.00mm x 1.00mm x 1.5mm. Additionally, a manufacturer-based dual-echo GRE sequence was used to acquire B0 field maps (voxel size = 4.00mm x 4.00mm x 5.00mm). A small number of subjects (n=11) were not able to be scanned at 3T due to pacemakers or other 3T-exclusionary criteria. For these subjects, a 1.5T Philips Intera system was used with a similar, adapted MRI protocol.  EPI DWI acquisitions are subject to substantial artifacts, including eddy current distortions and susceptibility- induced warping. Although these do not have a substantial impact on clinical assessment of large lesions associated with stroke or transient ischemic attack (TIA), they are quite large relative to the small embolic lesions resulting from the TAVI procedure – distortions may easily be on the order of 1cm, while lesions may be as small as a few mm. Therefore, a number of pre-processing steps were taken to improve image quality and subsequent analysis. First, the raw DWI images were corrected for eddy current induced distortions using the eddy correct tool from FMRIB’s FSL FDT library. Next, the diffusion b=0 (b0) and three corrected b=1000 diffusion-encoded raw images were combined to create trace and apparent diffusivity coefficient (ADC) images. Then, spatial distortions due to field susceptibility effects were corrected with the use of field maps. To accomplish this, the acquired B0 field maps were aligned to the individual T2-weighted DWI b0 images. Then, the FUGUE tool from FSL was used to perform map-based geometric unwarping.  Because the lesions are often so small, subtraction imaging was also employed to increase lesion salience.7 Baseline DWI and FLAIR images were voxel-wise subtracted from follow-up images to produce direct change maps. To facilitate this subtraction approach, additional pre-processing steps were performed. First, low-frequency spatial intensity inhomogeneities on FLAIR images were corrected using N3. Corrected FLAIR and DWI trace images were further standardized by applying a piecewise-linear histogram adjustment method to compensate for scan-to- scan variability in absolute intensity. Finally, to facilitate direct longitudinal analysis, all within-subject scans were co-registered to each subject’s baseline FLAIR image using FLIRT with 6 degrees of freedom.  Lesions were delineated on corrected and aligned 2 and 7 days DWI trace images using a semi-automated contouring technique provided by the JIM software package. Using this approach, a trained operator identified lesions individually, and for each lesion an assistive algorithm delineated a highly reproducible iso-contour at the maximum local gradient. The operator viewed all images and change maps simultaneously to increase confidence, and also coded lesions as new or persistent.  In addition to lesion counts and volumetry, vascular territory was also assessed using an atlas-based technique. For this purpose, a vascular territory atlas was manually created in the standard MNI 152 template space12 based on existing literature, and including 28 separate regions. Individual hires-T1 images were used to non-linearly align this atlas to individual lesion maps. First, individuals’ hires-T1 images were corrected for intensity inhomogeneity using N3, then aligned to the MNI 152 template using a two-stage process consisting of an initial rigid-body co- registration followed by composition with a warp field obtained from a non-linear warping technique. These transforms were then inverted, and applied to the original atlas. Lesion number and volume within each vascular territory were than assessed separately.  For visualization purposes, aggregate lesion maps were also rendered in 3D. First, individual subjects’ lesion maps were non-linearly aligned to MNI space as described above. Then, these individual maps were averaged within groups to produce group maps of lesion density. These were rendered using MayaVI, with regions with at least one lesion shown in yellow, and regions with two or more lesions in shown red. |
| Van Mieghem *et al.* | MISTRAL-C | No information was provided regarding use of a MRI core laboratory.  The MRI exam was performed with a 3.0 Tesla scanner with an 8-channel head coil. The MRI protocol consisted of three sequences: 1) transverse DW-MRI sequence with a b-value of 0,500,1000 s/mm2 (SE/EPI, TR 8,000 ms, TE 80 ms, FOV 24×24 cm, matrix 128×128, slice thickness 3.6 mm, 3 NEX); 2) sagittal 3D-FLAIR sequence (TR 6,500, TE 115, FOV 26×26 cm, matrix 224×224, slice thick- ness 1.2 mm, NEX 1); 3) 2D-T2w TSE sequence (TR 5,000 ms, TE 105 ms, FOV 24×24 cm, matrix 416×384, slice thickness 3 mm, NEX 2). The number, location, and volume (cm) of new hyperintense lesions were recorded. New lesions were allocated to the cerebellum, or the left or right vascular territory of the anterior, medial or posterior cerebral artery. To calculate the volume of hyperintense lesions on DWI, a semi-automated segmentation method was developed using MeVisLab (MeVis Medical Solutions AG, Bremen, Germany). The brain was arbitrarily divided into Sentinel CPS protected and unprotected regions. Unprotected regions are vulnerable to embolisations coming from the unprotected left vertebral artery, which corresponds to the cerebellum and the vascular territory of both posterior cerebral arteries. |
| Wendt *et al.* | EMBOL-X | No information was provided regarding use of a MRI core laboratory.  Magnetic resonance imaging was performed on a 1.5-T Avanto imaging system (Siemens, Erlangen, Germany). Patients were imaged before and after the procedure within 1 week. The protocol included the following 2 sequences: (1) transversal fluid-attenuated inversion recovery (repetition time, 9000 ms; echo time, 115 ms; matrix, 256 x 208; slice thickness, 6 mm); and (2) transversal diffusion-weighted images of the whole brain (repetition-time, 4,600 ms; echo-time, 137 ms; matrix, 128 x 128; slice-thickness, 5 mm; gradients of b volume, 0, 500, 1000 s/mm2). Diffusion images were processed to generate isotopic apparent diffusion coefficient maps with the use of the scanner’s dedicated software, allowing proper temporal classification of the lesions. Scans were evaluated for the presence of focal diffusion abnormalities in a pattern consistent with embolic lesions. The presence, number, volume, and location of all new focal diffusion abnormalities were recorded. |
| Lansky *et al.* | DEFLECT III | Diffusion-weighted magnetic resonance imaging of the brain was performed at 4 +/- 2 and 30 +/- 7 days post-procedure according to a standardized image acquisition protocol. Diffusion-weighted magnetic resonance imaging data were analysed at an independent core laboratory (Global Institute for Research, Richmond, VA, USA) by two independent imaging physicians using validated qualitative and quantitative methods (Vitrea Version 6.3.2, Toshiba America Medical Systems, Tustin, CA, USA) |

**Figure S1. Effects of cerebral embolic protection devices during transcatheter aortic valve implantation on total cerebral lesion volume in the protected brain.**

**
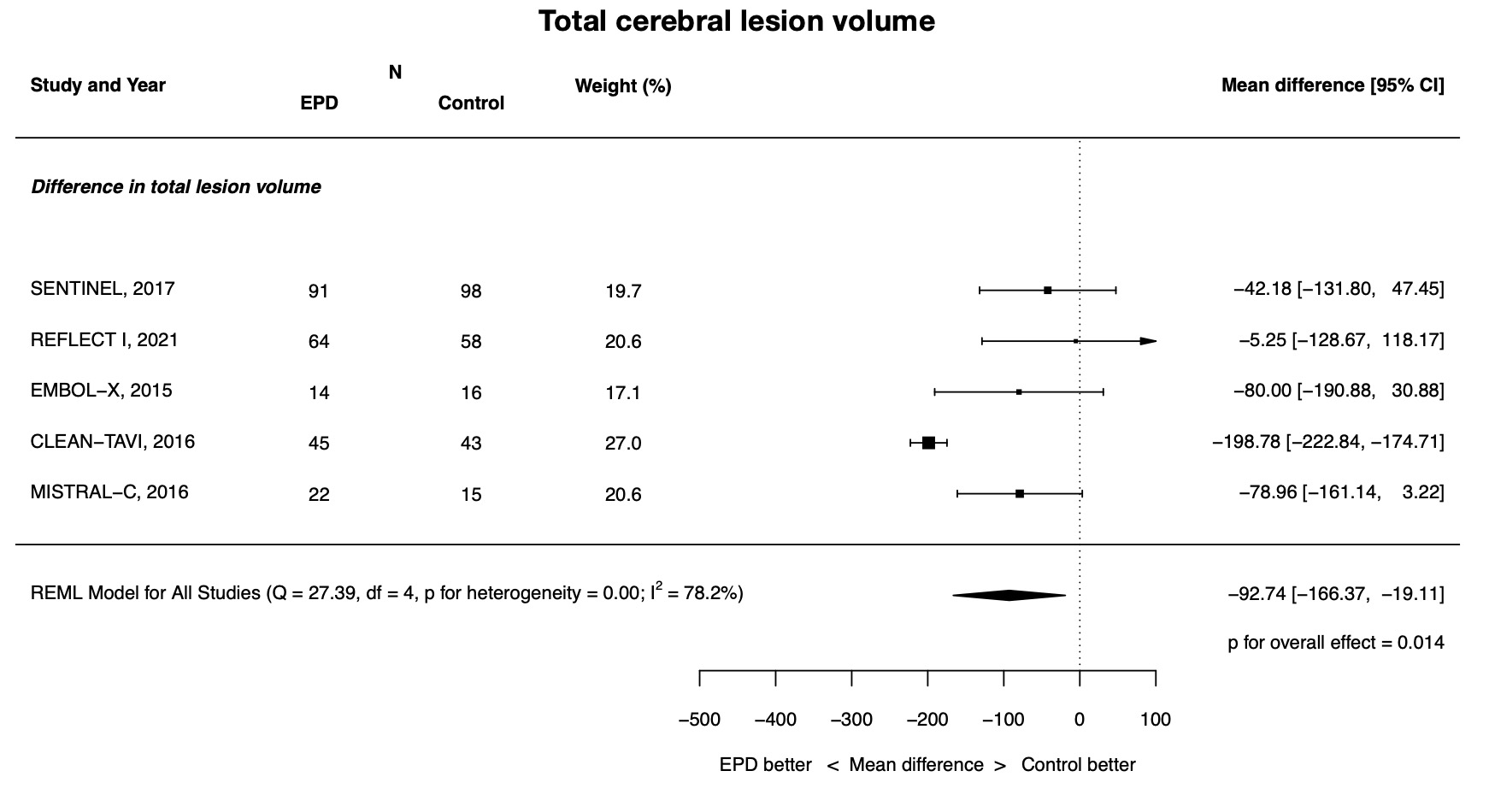
**
